# Allgemeinsymptome und Ansprechen auf Benzylpenicillin bei Erysipel und unkomplizierter Phlegmone – eine monozentrische, retrospektive, explorative Studie

**DOI:** 10.1111/ddg.15957_g

**Published:** 2026-06-04

**Authors:** Helena Schieffers, Cord Sunderkötter

**Affiliations:** ^1^ Universitätsklinik für Dermatologie und Venerologie Martin‐Luther‐Universität Halle‐Wittenberg, Halle (Saale); ^2^ MSB Medical School Berlin Hochschule für Gesundheit und Medizin, Berlin

**Keywords:** Allgemeinsymptomatik, Erysipel, Haut‐ und Weichgewebeinfektionen, Penicillin, unkomplizierte Phlegmone, Cellulitis, erysipelas, penicillin, skin and soft tissue infections

## Abstract

**Hintergrund:**

Erysipele gelten als durch Streptokokken verursachte Infektionen und sollen daher mit Penicillin behandelt werden, während Phlegmonen meist durch *Staphylococcus aureus* hervorgerufen werden und Penicillinase‐feste Betalaktamantibiotika benötigen. Letztere bergen ein höheres Risiko für unerwünschte Wirkungen. Daher ist die klinische Trennung dieser häufigen Weichgewebeinfektionen wichtig. Neben einem charakteristischen Erythem könnten hierfür die bei Erysipel oft angeführten Allgemeinsymptome hilfreich sein. Wir haben daher retrospektiv verglichen, wie häufig Patienten mit Erysipel im Vergleich zu Phlegmonen Allgemeinsymptome angegeben, und ob die Erysipele auf Penicillin angesprochen haben.

**Patienten und Methodik:**

Wir haben bei Patienten, die von 1.1.2024 bis 31.1.2025 in der Hautklinik der Universitätsmedizin Halle wegen Erysipels oder unkomplizierter Phlegmone stationär behandelt wurden (Entlassungsdiagnosen), die gemäß einem Standardverfahren erfassten Daten retrospektiv ausgewertet.

**Ergebnisse:**

Von 76 Patienten mit Erysipel gaben 91,4% an, zu Beginn oder bereits vor der Rötung Allgemeinsymptome bemerkt zu haben, darunter auch 17 von 18 Patenten mit Rezidiverysipel. Bei unkomplizierten Phlegmonen wurden Allgemeinsymptome nur bei 36,2% angegeben, teilweise erst im Verlauf und nie vor der Rötung. Patienten mit Erysipel sprachen zu 98,3% binnen 2 Tagen auf Benzylpenicillin an, darunter 21 Patienten, die vor Therapiebeginn bereits 4–10 Tage Symptome hatten.

**Schlussfolgerungen:**

Unsere Ergebnisse legen nahe, dass früh auftretende Allgemeinsymptome zu den charakteristischen Symptomen eines Erysipels zählen, und dass Erysipele keine schnelle Selbstheilungstendenz haben, aber auf Penicillin regelmäßig ansprechen.

## EINLEITUNG

Erysipel und unkomplizierte oder begrenzte Phlegmone^1^ gehören zu den weltweit häufigen Haut‐ und Weichgewebeinfektionen (HWGI).^2^, ^3^, ^4^ Es liegen keine genauen Registerdaten zu den Inzidenzen vor. Internationale Studien sind aufgrund bislang uneinheitlich verwendeter Definitionen nur eingeschränkt vergleichbar. Nach Angaben des Statistischen Bundesamtes wurden im Jahr 2016 etwa 70 000 Patienten mit der Diagnose Erysipel in Deutschland stationär behandelt.^5^

Trotz ihres häufigen Auftretens wird zwischen ihnen weder begrifflich noch klinisch oder therapeutisch immer klar genug unterschieden. Eine Abgrenzung ist jedoch bedeutsam für die Therapie: Erysipele können in der Regel allein mit Penicillin ausreichend behandelt werden, während Phlegmonen mindestens ein penicillinasefestes Betalaktamantibiotikum mit engem Spektrum, in einigen Fällen sogar ein breiter wirksames Antibiotikum erfordern.^1^, ^6^ Auch wenn diese Betalaktamantibiotika in der Regel gut verträglich sind, haben sie dennoch ein leicht höheres Risiko für Toxizitäten als Penicillin.^7^, ^8^, ^9^ Penicillin führt möglicherweise zu einer geringeren Änderung des Mikrobioms^10^ und wird in der Regel aufgrund seines engen Spektrums weniger resistente Erreger selektionieren als ein Antibiotikum mit breiterem Spektrum.^11^

Das klassische Erysipel wird als eine akute bakterielle, klinisch nicht eiternde Infektion der Dermis angesehen, die von beta‐hämolysierenden Streptokokken verursacht wird.^1^, ^12^, ^13^ Auch wenn deren kultureller Nachweis aus dem Gewebe oder aus Abstrichen meist nicht gelingt (zusammengefasst in^13^ und^1^, zudem Teilergebnis einer laufenden Untersuchung [Sunderkötter, Becker, et al., unveröffentlicht]), gilt das in Fallserien gezeigte Ansprechen auf Penicillin auch ohne Erregernachweis als indirekter Beleg für die Genese durch Streptokokken.^14^

Die unkomplizierte Phlegmone wird demgegenüber als eine nicht oder nur gering eiternde Infektion der Dermis, teilweise auch in die Subkutis reichend, definiert, die in der Regel durch *Staphylococcus (S.) aureus* verursacht wird.^1^ Sie stellt weder ein durch β‐hämolysierende Streptokokken bedingtes Erysipel noch eine eitrig‐nekrotisierende, bis an die Faszie reichende komplizierte Weichgewebeinfektion dar. In der ersten deutschsprachigen S2k‐Leitlinie zu HWGI wurden sie noch vorläufig als „begrenzte Phlegmonen“ bezeichnet,^1^ in der aktualisierten Fassung (bei Drucklegung noch nicht frei gegeben) jedoch als „unkomplizierte Phlegmonen“, da sich diese Bezeichnung begrifflich und definitorisch besser von den schweren, nun als „kompliziert“ bezeichneten Phlegmonen abgrenzen lässt.^15^ Letztere werden zusätzlich anhand der ursprünglich für klinische Studien entwickelten FDA‐Kriterien für „komplizierte Haut‐ und Weichgewebeinfektionen“ definiert.^16^

Die Abgrenzung des Erysipels von Phlegmonen erfolgt klinisch.^1^, ^17^ Die unkomplizierte Phlegmone ist charakterisiert durch eine überwärmte, teigige, oft druckdolente Schwellung mit dunkler oder livider Rötung, welche matter und unschärfer begrenzt ist als beim klassischen Erysipel und sich erfahrungsgemäß meist um ein Ulkus oder eine Wunde als Eintrittspforte herum bildet.^1^ Das Erysipel hingegen erscheint klinisch als akutes, überwärmtes, unterschiedlich schmerzhaftes, hellrotes Erythem mit glänzender Oberfläche, relativ scharf begrenzten, manchmal bogenförmig auslaufenden Rändern.^1^ Auf Grundlage von Fallberichten und Expertenmeinung werden als mögliches diagnostisches Kennzeichen neben diesen klinisch‐morphologischen Kriterien oft auch „Allgemeinsymptome“ genannt.^1^, ^18^ Gemeint damit sind Unwohlsein, Frösteln (selten Schüttelfrost) und Fieber als Zeichen einer systemischen Entzündungsreaktion. Es gab bislang aber keine Studie oder größere Fallserie, welche dieses Kriterium belegt. Fieber und Frösteln bildeten zwar vor allem in älteren französischen Studien ein Einschlusskriterium,^13^, ^18^, ^19^ doch in anderen Untersuchungen, in denen es speziell um Ursache oder Kennzeichen der Erysipele geht, werden sie nicht explizit besprochen,^20^ oder erwähnt.^14^, ^21^

Da an unserer Klinik seit 3 Jahren Patienten mit Weichgewebeinfektionen standardisiert nach Allgemeinsymptomen befragt, und bestimmte klinische Daten routinemäßig erfasst werden, wollten wir retrospektiv anhand der Patientenakten untersuchen, *(1)* wie häufig bei Patienten, bei denen aufgrund der klinischen Kriterien ein Erysipel oder eine unkomplizierte (begrenzte) Phlegmone diagnostiziert wurde, Allgemeinsymptome im Sinne von Fieber (≥ 38 °C), Frösteln oder Krankheitsgefühl angegeben wurden, *(2)* wie häufig Patienten mit Erysipel auf die in der Leitlinie empfohlene Penicillintherapie ansprachen, und *(3)* ob sich weitere Auffälligkeiten zeigten, die eine Unterscheidung zwischen Erysipel und unkomplizierter Phlegmone ermöglichen könnten.

## PATIENTEN UND METHODIK

In unsere retrospektive Kohortenstudie schlossen wir alle Patienten der Klinik für Dermatologie des Universitätsklinikums Halle ein, die von Januar 2024 bis Januar 2025 wegen eines Erysipels oder einer unkomplizierten (begrenzten) Phlegmone stationär behandelt wurden. Erfasst wurden sie über die jeweiligen Entlassungsdiagnosen. Patienten mit komplizierten Phlegmonen (komplizierten Haut‐ und Weichgewebeinfektionen, HWGI), nekrotisierenden HWGI oder Abszessen wurden ausgeschlossen.

Die Diagnose Erysipel wurde klinisch gestellt, wenn ein Erythem mit hellroter (statt livider) Farbe, mit eher glänzender Oberfläche und relativ scharfer Begrenzung vorlag und darunter keine teigige Schwellung oder Abszedierung bestand.

Die Diagnose Phlegmone wurde gestellt, wenn das Erythem lividrot und unscharf begrenzt war und darunter eine ödematöse oder teigige Schwellung bestand. Allgemeinsymptome oder Fieber schlossen die Diagnose nicht aus.

Nach dem Standardverfahren unserer Abteilung wurden das Vorhandensein oder Fehlen der für die Diagnostik relevanten Symptome dokumentiert. Darüber hinaus wurden routinemäßig folgende Parameter erfasst:

- mögliche Eintrittspforten,
- Schmerzhaftigkeit,
- anamnestische Angaben zu Allgemeinsymptomen wie Fieber, Frösteln oder Schüttelfrost, Unwohlsein, Abgeschlagenheit und Übelkeit (Tabelle 1). Hierzu wurden alle Patienten mit Weichgewebeinfektionen gemäß interner Arbeitsanweisung gezielt nach entsprechenden Symptomen befragt (zum Beispiel: „Fühlten Sie sich kurz vor oder nach Auftreten der Rötung wie bei einer beginnenden Erkältung, haben Sie gefröstelt oder Schüttelfrost beziehungsweise Fieber gehabt oder fühlten Sie sich abgeschlagen?“),
- anamnestische Angaben zur Dauer der Symptome vor Vorstellung und vor Beginn der Therapie,
- chronisches Lymphödem oder Lipolymphödem im Bereich der Infektion,
- Entzündungsparameter: C‐reaktives Protein (CRP) und Blutbild,
- weitere bei Aufnahme bestehende akute Infektionen, die Allgemeinsymptome verursachen oder bei der Auswahl der Antibiotikatherapie berücksichtigt werden mussten.

**TABELLE 1:** Erfragte Allgemeinsymptome (Mehrfachnennung war möglich).

Angegebene Allgemeinsymptome bei Erysipel (n = 76)	
Fieber	39 (51,3%)
Frösteln (teils von Patienten als „Schüttelfrost“ angegeben)	32 (42,1%)
Nachtschweiß	2 (2,6%)
Abgeschlagenheit und reduzierter Allgemeinzustand	34 (44,7%)
Übelkeit / Erbrechen	9 (11,8%)
Gliederschmerzen	2 (2,6%)

Zur Behandlung des Erysipels wurde Penicillin G eingesetzt; die Standarddosierung betrug 10 Mio. I.E. dreimal täglich. Bei anamnestisch geäußertem oder bestätigtem Verdacht auf eine Penicillinallergie war als erste Alternative Cefazolin 2 g dreimal täglich unter Notfallbereitschaft vorgesehen.^22^ Patienten mit der Diagnose Phlegmone erhielten leitliniengerecht eine parenterale Therapie mit Cefazolin (1‐2 g dreimal täglich). Bei ausgedehnter Infektion oder ausgeprägter Paraklinik wurde Ampicillin/Sulbactam (2 g/1 g dreimal täglich) verabreicht oder – bei zusätzlichen Infektionsherden, etwa komplizierten Harnwegsinfekten – Cefuroxim (1,5 g dreimal täglich).

Ein Ansprechen auf die Therapie wurde gemäß interner Arbeitsanweisung dokumentiert, wenn folgende Kriterien erfüllt waren:

- Bei Erysipel: Auftreten einer feinen Fältelung der Haut über dem Erythem (Hinweis auf Rückgang der subepidermalen Ödeme) und/oder flächenmäßiger Rückgang beziehungsweise Abblassen der Rötung.
- Bei Phlegmone: Flächenmäßige Reduktion der Rötung und/oder Rückgang der teigigen Schwellung unter dem Erythem beziehungsweise Abblassen der Rötung.
- Bei beiden Entitäten zusätzlich: Rückgang eventueller Allgemeinsymptome und Schmerzen, Rückgang des CRP‐Werts (um mindestens 25% nach etwa 3 Tagen) oder der Leukozytenzahl (frühestens nach 24 Stunden; absolute Werte, kein prozentualer Mindestwert)

### Ethikvotum

Die retrospektive Analyse der Daten wurde mit Zustimmung der lokalen Ethikkommission (Medizinische Fakultät der Martin‐Luther‐Universität Halle‐Wittenberg durchgeführt (0205‐022).

## ERGEBNISSE

Zwischen dem 01.01.2024 und dem 31.01.2025 wurden im Archiv unserer dermatologischen Klinik insgesamt 123 Patienten mit den Entlassungsdiagnosen Erysipel (n = 76) oder Phlegmone (n = 47) gefunden. In fünf Fällen war eine initiale Diagnose „Erysipel“ im Verlauf des Aufenthaltes in Phlegmone (Entlassungsdiagnose) korrigiert worden, weil eine ausgeprägte oder teigige lokale Schwellung bestand (sie alle hatten Allgemeinsymptome); diese Patienten wurden in dieser Analyse entsprechend als Phlegmone eingruppiert (Tabellen S1, S2 im Online‐Supplement).

### Klinisches Bild

**ABBILDUNG 1:**
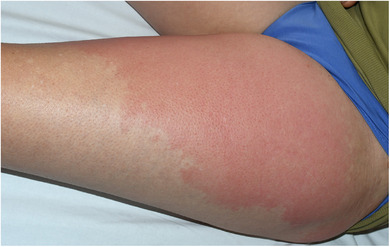
Klassisches Erysipel am Oberschenkel mit bogigen (flammenförmigen) Ausläufern.

**ABBILDUNG 2:**
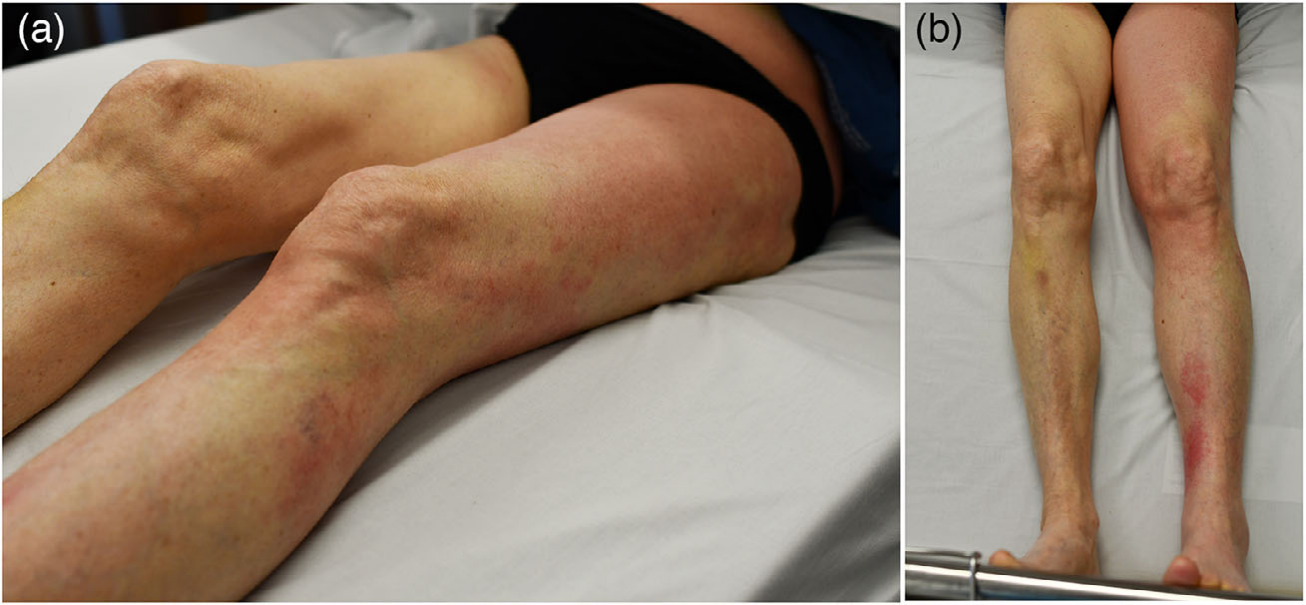
Erysipel am Bein mit unterbrochener, inselartiger Verteilung der Rötung bei chronischem Lymphödem des linken Beines. (a) Nahaufnahme, seitlich. (b) Übersicht, frontal auf der das Lymphödem im Vergleich zum rechten Bein gut zu erkennen ist.

**ABBILDUNG 3:**
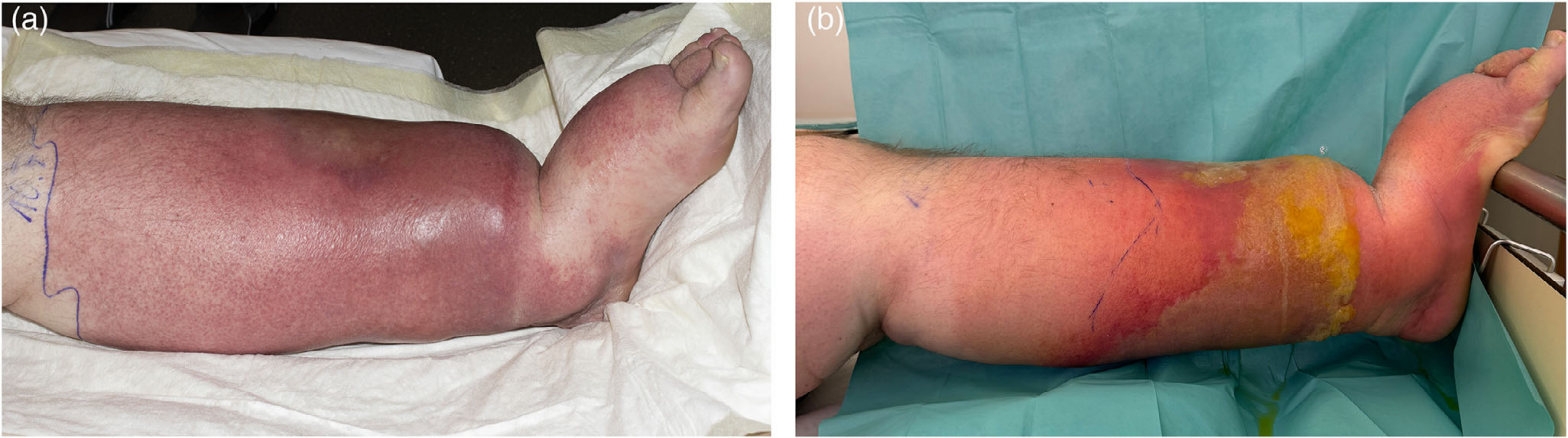
Erysipel bei chronischem Lymphödem mit zentraler Blasenbildung. (a) Tag 2 mit beginnender Blasenbildung. (b) Tag 6, die Blase hat sich großflächig mit serösem, gelblichen, aber nicht eitrigen Exsudat gefüllt, wähend das Erythem teilweise abgeblasst ist.

Bei den Patienten mit Erysipel ist in 70 Fällen (92,1%) ein durchgehend hellrotes oder flammend rotes Erythem beschrieben worden (Abbildung 1), bei sechs Patienten (7,9%) wurden zusätzlich im hellroten Erythem livide Anteile beschrieben, von denen in fünf Fällen das Erysipel auf einem chronischen Lymphödem aufgetreten war und hier die sonst ganzheitlich flächige Rötung teilweise in einzelne, oft nur über einen schmalen Streifen verbundene Erytheme inselartig aufgebrochen war (Abbildung 2a, b). Auf neun der anderen Erysipele wurden zusätzlich serös gefüllte oder hämorrhagische Blasen (n = 6) und/oder Einblutungen (n = 4) beschrieben (das gelblich‐seröse Exsudat nahm mitunter im Verlauf zu, nachdem die Rötung bereits auf die Therapie angesprochen hatte) (Abbildung 3a, b).

**ABBILDUNG 4:**
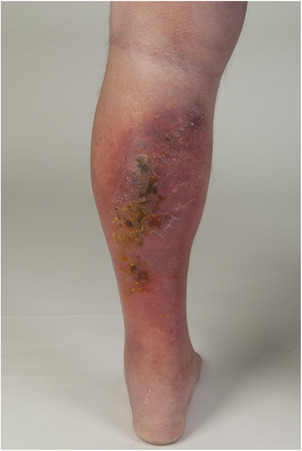
Unkomplizierte Phlegmone des Unterschenkels mit unscharfer Begrenzung und vorwiegend lividem Colorit.

Bei den Patienten mit der Diagnose unkomplizierte oder begrenzte Phlegmone ist in 33 von 47 Fällen (70,2%) auf einer unterschiedlich ausgeprägten teigigen Schwellung ein rotes, in 13 Fällen (27,7%) ein rotlivides Erythem beschrieben worden (Abbildung 4), ein zusätzlicher Anteil mit hellrotem Kolorit wurde nur in einem Fall (2,1%) beschrieben, hier lag eine unscharfe Begrenzung vor. Die meisten Erysipele und Phlegmonen befanden sich an den Unterschenkeln (Tabelle 2).

**TABELLE 2:** Betroffene Hautareale bei Erysipel und unkomplizierter (begrenzter) Phlegmone.

Lokalisation	Erysipel (n = 76)	Phlegmone (n = 47)
Ganzes Bein	5 (6,6%)	1 (2,1%)
Oberschenkel/Leiste	2 (2,6%)	5 (10,6%)
Unterschenkel	53 (69,7%)	18 (38,3%)
Fuß	2 (2,6%)	2 (4,3%)
Gesicht	14 (22,4%)	9 (19,1%)
Arm	2 (2,6%)	4 (8,5%)
Hand	0	3 (6,4%)
Mamma	1 (1,3%)	0
Gluteal/Rücken	0	2 (4,3%)
Unterbauch	0	1 (2,1%)
Axilla bei Lymphozele	0	1 (2,1%)
Capillitium	0	1 (2,1%)

### Allgemeinsymptome

Von den Patienten mit Erysipel gaben 92,1% (70 von 76) an, dass sie mit oder noch vor Bemerken des Erythems Allgemeinsymptome im Sinne von Fieber, Schüttelfrost, Frösteln gehabt und sich abgeschlagen oder krank gefühlt hätten (Tabelle 1). Darunter waren auch 17 von 18 Patienten mit rezidivierendem Erysipel (14 mit Fieber oder Schüttelfrost [2 trotz Rezidivprophylaxe], 3 mit Abgeschlagenheit). Neun gaben zusätzlich Übelkeit und Erbrechen an (Tabelle 1). Fast alle (n = 66) empfanden diese Symptome als deutlich und beeinträchtigend. Nach der stationären Aufnahme wurde bei sechs Patienten eine zusätzliche Infektion festgestellt. In vier Fällen bestanden ein ansonsten asymptomatischer Harnwegsinfekt und in zwei Fällen eine Bronchopneumonie. Auch wenn die Allgemeinsymptome zeitlich mit dem Auftreten der Rötung zusammenfielen und somit zumindest für die häufig asymptomatisch verlaufenden Harnwegsinfekte nicht typisch wären, wurden diese Fälle bei der Berechnung des Anteils der Erysipele mit Allgemeinsymptomen nicht berücksichtigt. Der Anteil betrug demnach 91,4 % (64 von 70).

Von den sechs Patienten ohne dokumentierte Allgemeinsymptomatik standen zwei unter einer Dauertherapie mit Metamizol, eine Patientin befand sich nach Radiatio bereits in deutlich reduziertem Allgemeinzustand, und zwei Patienten erhielten bereits eine orale Antibiotikatherapie (letztere verhinderte allerdings nicht immer Allgemeinsymptome, weil fünf von sieben weiteren Patienten mit bestehender oraler Antibiotikatherapie Allgemeinsymptome angegeben hatten). Die Angabe, dass die Allgemeinsymptomatik mitunter bereits 1–2 Tage vor der Rötung bemerkt wurde, hatte dazu geführt, dass wir ab Oktober 2024 diesen Umstand zusätzlich ausdrücklich bei jedem Patienten erfragt und dokumentiert haben. Danach wurde bei zwölf von 20 Fällen mit Erysipel vermerkt, dass Frösteln oder Fieber und Abgeschlagenheit bereits vor (n = 7) oder zusammen mit (n = 5) einer noch flächenmäßig kleinen Rötung aufgefallen waren. Bei einigen Patienten waren die Allgemeinsymptome zum Zeitpunkt der Vorstellung sogar schon mehr oder minder zurückgegangen.

Demgegenüber gaben von den Patienten mit unkomplizierter Phlegmone nur 36,2% (n = 17) Allgemeinsymptome an, meist Fieber und Schüttelfrost, zwei dieser Patienten hatten zuvor eine Antibiotikatherapie erhalten. In sieben Fällen wurden sie gleichzeitig mit der Rötung bemerkt, in fünf Fällen seien sie erst aufgetreten, nachdem Rötung oder Schwellung zugenommen hätten und in fünf Fällen fehlten hierzu Angaben. In keinem Fall war ein Auftreten von Allgemeinsymptomen vor Bemerken der Hautveränderung dokumentiert worden, obwohl auch diese Patienten seit Oktober explizit danach gefragt worden waren. Bei den Patienten ohne Allgemeinsymptome war keine dauerhafte Gabe von Antiphlogistika dokumentiert worden.

### Schmerzen

Schmerzen wurden bei 47 der Patienten mit Erysipel (59,5%) angegeben, die anderen 34 Patienten (43%) hatten sie verneint. Drei Patienten klagten im Bereich des Erythems über punktuell starke, bei Druck zunehmende Schmerzen. Eine Thrombose konnte jeweils ausgeschlossen werden, jedoch zeigte sich im Ultraschall am Punkt maximaler Schmerzempfindung ein deutliches Ödem in einzelnen Bindegewebssepten. Bei Phlegmonen hatten 27 Patienten (57,4%) Schmerzen bejaht, 18 Patienten (38,3%) verneint, bei zwei Patienten fand sich keine Angabe.

### Potenzielle Eintrittspforten

Bei Erysipelen wurde in 50 Fällen (68,5 %) eine Mazeration oder Erosion (meist Interdigitalmykose) beschrieben, in vielen weiteren Fällen war keine und nur selten eine größere Eintrittspforte gesehen worden (Tabelle 3). Aus entsprechenden Abstrichen konnten nicht immer Bakterien isoliert werden, und wenn, dann *S. aureus* oder Streptokokken (unveröffentlichte Daten).

**TABELLE 3:** Mögliche Eintrittspforten bei Erysipel und unkomplizierter (begrenzter) Phlegmone.

Oberflächliche Defekte		
	*Erysipel*	*Phlegmone*
Mazeration, Mykose, Erosion	50	7
Keine Eintrittspforte ersichtlich	21	4
Impetiginisierte Erosionen	0	6
Follikulitis	0	2
Rhagade	0	1
Erosion bei Calcinosis cutis	0	1
*Gesamt*	71 (93,4%)	21 (44,7%)

Tiefe Defekte		
	*Erysipel*	*Phlegmone*
Ulcus	3 (flache Ulzera)	9 (davon 1 Decubitus)
Frische Tätowierung	1	0
OP‐Wunde	1	5
(Schnitt‐)Wunde	0	3
Abszess	0	4
Manipulierte Zyste	0	2
Furunkel	0	1
Lymphozele, Z. n. Punktion	0	1
Bursitis	0	1
*Gesamt*	5 (6,6%)	26 (56,5%)

Bei den Phlegmonen wurden oft Ulzera (19,1%) oder Abszesse (14,9%) oder OP‐Wunden dokumentiert (Tabelle 3). Aus Abstrichen konnten verschiedene gramnegative Bakterien und fast immer *S. aureus* kultiviert werden (unveröffentlichte Daten).

### Laborparameter

Patienten mit der Diagnose Erysipel wiesen bei Aufnahme durchschnittliche CRP‐Werte von 81,49 mg/dl (Bereich 3–219,6 mg/dl; Standardabweichung [SD] 77,2) und Leukozytenzahlen von 11,74 × 10^9^/l (Bereich 4,1–25 × 10^9^/l; SD 5,34) auf. Bei Patienten mit Phlegmone lagen die CRP‐Werte im Mittel bei 95,88 mg/dl (Bereich 2,8–451 mg/dl; SD 110,26) und die Leukozytenzahlen bei 12,73 × 10^9^/l (Bereich 5,1–43,8 × 10^9^/l; SD 5,80). Aufgrund der großen Streuung sind die Werte nur eingeschränkt interpretierbar. Zwischen Erysipel und unkomplizierter Phlegmone bestand kein signifikanter Unterschied der CRP‐Werte oder Leukozytenzahlen.

### Therapie und Ansprechen

Von den Patienten mit Erysipel wurden 60 von 76 mit Penicillin behandelt; zwei davon erhielten aufgrund hämorrhagischer Blasen zusätzlich kurzfristig Clindamycin zur Hemmung der Toxinsynthese. Bei sechs Patienten wurde wegen einer anamnestisch angegebenen oder im Allergieausweis vermerkten Unverträglichkeit von Betalaktam‐Antibiotika oder Penicillinallergie entsprechend der klinikinternen Arbeitsanweisung Cefazolin unter Notfallbereitschaft gegeben. Drei Patienten erhielten bei Verdacht auf eine zusätzliche staphylogene (Super‐)Infektion ebenfalls Cefazolin. Die sechs Patienten mit einer zweiten Infektion (Pneumonie oder Harnwegsinfekt) wurden nach Diagnosestellung mit Ampicillin/Sulbactam, Cefuroxim oder Ceftriaxon (alle i.v.) behandelt. Eine Patientin entwickelte erst später während des stationären Aufenthaltes eine Aspirationspneumonie und wurde daraufhin auf Ampicillin/Sulbactam umgestellt. Fünf Patienten hatten zuvor orale Antibiotika erhalten, aber darauf nicht angesprochen (2 x Penicillin V, 1 x Penicillin V und Azithromycin, 1 x Clindamycin, und 1 x ein nicht mehr eruierbares Antibiotikum).

Von den 60 mit Penicillin behandelten Patienten sprachen 59 Patienten (98,3%) binnen 2 Tagen an, im Sinne einer Fältelung der Haut, gefolgt von einem Abblassen und dann einer flächenmäßigen Reduktion der Rötung, begleitet von einem Rückgang noch bestehender Allgemeinsymptome. In einem Fall wurde aufgrund von unzureichendem klinischem Ansprechen und ausbleibendem Rückgang des CRP nach 3 Tagen die antibiotische Therapie um Clindamycin erweitert, hierunter kam es zu einem Ansprechen.

Von den mit Penicillin behandelten Patienten bestanden bei elf Patienten die Symptome bereits mehr als 6 Tage und bei zehn Patienten über mindestens 4 bis 6 Tage, bevor die Therapie begonnen wurde, und ohne dass bei diesen Patienten weitere begleitende Infektionen festgestellt worden waren. Sie sprachen dann aber alle binnen 2 Tagen auf die Antibiotikatherapie an.

Von den 47 Patienten mit der Diagnose Phlegmone hatten 36 Cefazolin, drei Ampicillin/Sulbactam, zwei Clindamycin und einer Cefuroxim erhalten. Die Patienten, die zuvor bei Aufnahmediagnose Erysipel Penicillin erhalten und darauf nicht ausreichend angesprochen hatten, erhielten Cefazolin (n = 3) oder Ampicillin/Sulbactam (n = 2). In sechs Fällen wurde bei unzureichendem Ansprechen nach 2 Tagen das Antibiotikum umgestellt von Cefazolin auf Ampicillin/Sulbactam, die übrigen Fälle sprachen innerhalb von 2–3 Tagen auf die Therapie an im Sinne eines Rückganges der geröteten Fläche und der teigigen Schwellungen unter dem Erythem sowie eines Abblassens der Rötung. Nur zwei Patienten hatten bereits vorher ambulant oral Antibiotika erhalten (Penicillin V und Amoxicillin/Clavulansäure), worunter es zu keinem Rückgang der Beschwerden gekommen war.

## DISKUSSION

In der von uns untersuchten Kohorte zeigte sich, dass *(1)* bei über 90 % der Patienten mit einem Erysipel Allgemeinsymptome aufgetreten waren, alle zu Beginn der Infektion, *(2)* bei unkomplizierter Phlegmone hingegen nur in 36% der Fälle Allgemeinsymptome angegeben wurden und teilweise erst im Verlauf, sowie dass *(3)* bei Vorliegen dieser Symptome beim Erysipel die Therapie mit Penicillin wirksam war. Aufgrund der hier zugrunde liegenden Daten ließe sich folgern, dass neben dem charakteristischen Erythem eine anfängliche Allgemeinsymptomatik ein entscheidendes Kriterium für die klinische Diagnose des Erysipels darstellt.

Die Allgemeinsymptome waren auch bei allen den Patienten neu im Zusammenhang mit dem Erythem aufgetreten, bei denen nach Aufnahme zusätzlich eine bereits bestehende weitere Infektion, meist ein ansonsten asymptomatischer Harnwegsinfekt, festgestellt wurde. Bei den sechs Patienten ohne dokumentierte Allgemeinsymptome könnten eine Therapie mit Metamizol und eventuell auch mit Antibiotika die Allgemeinsymptome unterdrückt oder früh verhindert haben, bei einer weiteren Patientin war ein nach Bestrahlung geschwächter Zustand in der Akte festgehalten worden. Somit könnte bei fünf der sechs Patienten ein möglicher Grund vorgelegen haben, warum Allgemeinsymptome nicht angegeben oder bemerkt wurden. Zu den Allgemeinsymptomen mögen auch Übelkeit und Erbrechen gehören, die aber nur zusammen mit einem der anderen, oben genannten Symptomen aufzutreten scheinen.

Bei unkomplizierten Phlegmonen traten Allgemeinsymptome hingegen nicht nur deutlich seltener (36,2%) auf, sondern mitunter auch ausdrücklich erst im Verlauf der Infektion; in unserer Kohorte jedenfalls wurde, anders als beim Erysipel, in keinem Fall von Allgemeinsymptomen berichtet, die vor Bemerken der Rötung aufgetreten seien.

Auch wenn Erysipele aufgrund ihres hellroten, oft leicht glänzenden und bogigen Erythems klinisch meist gut von der lividroten, teigigen Schwellung der unkomplizierten Phlegmone unterschieden werden können, würde die Berücksichtigung von Allgemeinsymptomen eine zusätzliche differenzialdiagnostische Sicherheit verleihen, und offenbar vor allem dann, wenn diese zu Beginn auftreten. Es kann unserer Erfahrung nach daher sinnvoll sein, die Patienten mit einer Weichgewebeinfektion nicht nur nach vorliegenden Allgemeinsymptomen zu fragen, sondern auch explizit danach, ob solche Allgemeinsymptome zu Beginn der Erkrankung bemerkt wurden. Aufgrund ihres frühen und offenbar mitunter vorübergehenden Auftretens könnte es sein, dass sie nicht aktiv benannt oder gar nicht mit der danach bemerkten Rötung in Zusammenhang gebracht wurden. Wenn die entsprechende Frage nicht Teil unserer einschlägigen Anamnese gewesen wäre, wäre diese initiale Allgemeinsymptomatik wahrscheinlich weniger häufig in der Krankenakte dokumentiert worden. Hierin mag begründet sein, warum Allgemeinsymptome nicht immer als besonderes Kriterium herausgearbeitet wurden,^20^ auch wenn Fieber und Frösteln zum Beispiel in französischen Studien als Einschlusskriterien für *erysipelas/cellulitis* genannt, aber ansonsten nicht gesondert erörtert wurden.^13^, ^19^

Zu den möglichen Ursachen dieser frühen Allgemeinsymptome liegen bislang keine entsprechenden Untersuchungen vor. Hinweise ergeben sich jedoch aus gut konzipierten systematischen Studien zur akuten Streptokokken‐Pharyngitis: Wenn es nach Einbringen von Streptokokken der Gruppe A zu einer manifesten Infektion kam, wurde ein deutlicher Anstieg unter anderem des Interleukin‐1‐Rezeptorantagonisten (IL‐1Ra) und von Interleukin 18 (IL‐18) gemessen.^23^ Diese Zytokine tragen direkt oder indirekt zu Fieber und anderen Entzündungszeichen bei und werden über einige der 13 Superantigene sowie über weitere Virulenzfaktoren der Streptokokken induziert.^24^

Ein weiterer auffälliger Unterschied zwischen Erysipel und unkomplizierter Phlegmone bestand in der Art der möglichen Eintrittspforte: Bei Phlegmonen war meist eine größere, auch tiefere Hautschichten einbeziehende Läsion dokumentiert, während bei Erysipelen häufig keine eindeutige Eintrittspforte oder lediglich kleinere Erosionen, meist Mazerationen im Rahmen einer Zehenzwischenraum­mykose, beschrieben wurden.

Die CRP‐Werte und Leukozytenzahlen waren in unserer Kohorte sowohl bei Erysipel als auch bei Phlegmone häufig und unterschiedlich stark erhöht. Im Gegensatz zu einer anderen retrospektiven Studie (unter Mitwirkung von C.S.)^25^ konnten wir keine signifikant höheren Werte bei Erysipelen feststellen. Die Aussagekraft unserer Ergebnisse ist jedoch aufgrund der großen Varianz eingeschränkt. Ein möglicher Grund hierfür könnte darin liegen, dass sich die Patienten in unterschiedlichen Krankheitsstadien vorstellten, teils erst, nachdem die Symptome bereits über einen gewissen Zeitraum bestanden hatten.

Die Morphe des hellroten Erythems mit teilweise scharf begrenzten, auch bogigen Rändern war ein differenzierendes Einschlusskriterium. Auffällig war, dass die ansonsten homogene Rötung mitunter inselartig aufgebrochen und nicht gleichmäßig hellrot erschien, meist dann, wenn das Erysipel auf einem chronischen Lymphödem auftrat. Auffallend war zudem, dass sich bei Aufnahme vorhandene seröse Blasen im Verlauf gelegentlich mit gelblich‐serösem Exsudat füllten, während die Rötung bereits auf die Therapie angesprochen hatte. Dieser Verlauf stellt somit kein Zeichen eines Progresses dar; der seröse Inhalt darf nicht fälschlicherweise als Eiter interpretiert werden.

Ausgeprägte akute Ödeme unter dem Erythem sind für Erysipele untypisch – außer, wenn sie im Gesicht oder Genitalbereich auftreten, wo die lockere Textur des Bindegewebes die Entwicklung von Ödemen erleichtert. In diesen Fällen ist eine Unterscheidung zur Phlegmone schwierig. Das Fehlen initialer Allgemeinsymptome könnte somit zum entscheidenden Kriterium für die Diagnose einer Phlegmone und gegen ein Erysipel werden. Aufgrund unserer Ergebnisse wäre es in der Tat gerechtfertigt, Frösteln, Fieber, Schüttelfrost und/oder Abgeschlagenheit zu Beginn einer Infektion als ein nahezu notwendiges, wenn auch nicht ausreichendes Kriterium für die Diagnose Erysipel anzusehen. Die Bestätigung dieser Annahme könnte durch eine prospektive, möglichst multizentrische Erhebung erfolgen, die zudem genauer und breiter erfassen würde, *(1)* wie häufig Allgemeinsymptome bereits vor oder unmittelbar mit dem Auftreten des Erythems beobachtet werden, *(2)* wie lange sie anhalten oder wie oft sie noch vor Therapiebeginn wieder abklingen, sowie gegebenenfalls *(3)* ob sie mit erhöhten Spiegeln von IL‐1Ra oder IL‐18 einhergehen. Durch den Einbezug unkomplizierter Phlegmonen ließe sich darüber hinaus prospektiv untersuchen, in welchem Ausmaß diese Symptome zu Beginn einer Phlegmone seltener auftreten und wie häufig sie erst im Verlauf entstehen.

Zur Therapie der im klinischen Alltag der Dermatologie, Allgemeinmedizin und Pädiatrie häufigen Erysipele oder unkomplizierten Phlegmonen werden laut einer Metaanalyse immer noch zu oft Antibiotika mit breitem Spektrum und entsprechenden nachteiligen Folgen eingesetzt.^26^ Stattdessen genügen die seit Jahren verfügbaren Antibiotika mit engem Wirkspektrum, die auch ohne aktuelle randomisierte Studien weiterhin empfohlen werden.^6^, ^15^ Für das Erysipel gilt Penicillin (zum Beispiel Benzylpenicillin i.v. 3 × 10 Mio. IU/Tag für 7 Tage) als Mittel der Wahl, auch wenn in entsprechenden Fallserien nicht immer klar zwischen Erysipel und unkomplizierter Phlegmone unterschieden wurde.^12^, ^27^ Unsere retrospektive Erfassung bestätigt mit einer Ansprechrate von 98,3% die Wirksamkeit von Penicillin bei Erysipel. Die zusätzliche Gabe von Clindamycin in einzelnen schweren Fällen (n = 2) mit hämorrhagischen Blasen oder Seropapeln beruht auf Expertenempfehlung und leitet sich aus einer Studie ab, der zufolge die Hemmung der bakteriellen Protein‐ und damit auch Toxinsynthese durch eine dreitägige Clindamycintherapie bei toxinvermittelten, nekrotisierenden Weichgewebeinfektionen zu einer Senkung der Mortalität führte.^28^

Unsere Beobachtung zur Notwendigkeit einer Behandlung zeigt, dass viele Patienten bereits seit mehreren Tagen ein Erythem hatten, bevor sie ärztliche Hilfe suchten, dann jedoch innerhalb von zwei Tagen deutlich auf die Therapie ansprachen. Dies deutet darauf hin, dass das Erysipel – trotz des häufig nicht gelungenen Erregernachweises – keine schnelle spontane Rückbildungstendenz zeigt, sondern einer gezielten Penicillintherapie bedarf.

Die Patienten mit Phlegmonen sprachen zu 83,3 % auf die Therapie mit einem penicillinasefesten Betalaktamantibiotikum mit engem Wirkspektrum (Cefazolin) an; in den übrigen Fällen zeigte sich ein Ansprechen auf die in der Leitlinie als nächste Option empfohlenen Medikamente.^6^, ^15^

Auch wenn diese Betalaktamantibiotika in der Regel gut verträglich sind, ist ihr Risiko für unerwünschte Wirkungen im Vergleich zu Penicillin höher.^7^ Dazu zählen zum Beispiel eine durch einen Metaboliten verursachte Leberschädigung bei Flucloxacillin,^29^ Kristallurie und Nierenversagen unter hochdosierter parenteraler Gabe von Ampicillin oder Amoxicillin^30^ sowie die lebertoxische Wirkung von Clavulansäure. Im direkten Vergleich mit Penicillin zeigten Oxacillin, Nafcillin^8^ und – in etwas geringerem Ausmaß – auch Cefazolin^9^ häufiger unerwünschte Wirkungen. Sie werden daher nicht als Mittel der ersten Wahl zur Behandlung des Erysipels empfohlen.^15^ Auch unsere retrospektive Analyse legt nahe, dass zur Therapie des Erysipels kein anderes Betalaktamantibiotikum als Penicillin erforderlich ist.

Mögliche Einschränkungen unserer Ergebnisse ergeben sich aus dem retrospektiven Studiendesign, dem Einschluss ausschließlich stationärer Patienten, der Erhebung und Dokumentation der Parameter durch verschiedene Ärzte unserer dermatologischen Abteilung sowie einer potenziellen Voreingenommenheit hinsichtlich der Bedeutung der Allgemeinsymptome. Gegen eine ausgeprägte Verzerrung spricht jedoch, dass Allgemeinsymptome auch bei einigen unkomplizierten Phlegmonen dokumentiert wurden, dass bei fünf Patienten mit der Entlassungsdiagnose Phlegmone die ursprüngliche Diagnose – trotz angegebener Allgemeinsymptome – von Erysipel auf Phlegmone korrigiert wurde und dass die Ansprechrate auf Penicillin bei als Erysipel eingestuften Fällen nahezu 100 % betrug. Letzteres wäre aufgrund der Penicillinase‐bildenden S. aureus nicht zu erwarten gewesen, wenn tatsächlich eine Phlegmone irrtümlicherweise als Erysipel diagnostiziert worden wäre.

## DANKSAGUNG

Open access Veröffentlichung ermöglicht und organisiert durch Projekt DEAL.

## INTERESSENKONFLIKT

C.S. hat im Zusammenhang mit Weichgewebeinfektionen Honorare für Vorträge und Beratungstätigkeiten von Bayer AG, Correvio, Infectopharm, GlaxoSmithKline und Novartis erhalten. H.S. erklärt, dass kein Interessenkonflikt besteht.

## Supporting information



Supplementary information
